# Analyse einer differenzierten Schockraumalarmierung an einem überregionalen Traumazentrum

**DOI:** 10.1007/s00113-023-01391-0

**Published:** 2023-11-20

**Authors:** Jonas Limmer, Mila M. Paul, Martin Kraus, Hendrik Jansen, Thomas Wurmb, Maximilian Kippnich, Daniel Röder, Patrick Meybohm, Rainer H. Meffert, Martin C. Jordan

**Affiliations:** 1 Klinik und Poliklinik für Unfall‑, Hand‑, Plastische und Wiederherstellungschirurgie, Universitätsklinikum Würzburg, Oberdürrbacher Str. 6, 97080 Würzburg, Deutschland; 2 Regierung von Unterfranken, Stephanstr. 2, 97070 Würzburg, Deutschland; 3 Klinik und Poliklinik für Anästhesiologie, Intensivmedizin, Notfallmedizin und Schmerztherapie, Universitätsklinikum Würzburg, Oberdürrbacher Str. 6, 97080 Würzburg, Deutschland

**Keywords:** Polytrauma, Unfallchirurgie, Traumanetzwerk, Schwerstverletztenversorgung, Übertriage, Polytrauma, Trauma surgery, Trauma network, Seriously injured care, Overtriage

## Abstract

**Hintergrund:**

Um die sowohl personal- als auch ressourcenintensive Versorgung verunfallter Patienten effizient zu gestalten, wurden in einigen Krankhäusern unterschiedliche Abstufungssysteme hinsichtlich der Schockraumalarmierung eingeführt. Ziel dieser Arbeit war es, an einem ÜTZ in Bayern das Konzept von Schockraum A und B hinsichtlich der Praktikabilität, Indikationsstellung und möglicher Komplikationen zu evaluieren.

**Methodik:**

In einer retrospektiven Studie wurden Daten des Kollektivs von traumatischen Schockraumpatienten des Jahres 2020 erhoben. Die Zuteilung in A und B erfolgte durch den präklinischen Notarzt. Es wurden hierbei die Parameter ISS, GOS, Upgrade-Rate sowie die Indikationskriterien nach damals geltender S3-Leitlinie erhoben. Die statistischen Datenvergleiche erfolgten mittels *t*-Test, χ^2^ oder Mann-Whitney‑U Test.

**Ergebnisse:**

Insgesamt erfüllten 879 Schockräume (A: 473, B: 406) die Einschlusskriterien. Hierbei zeigte sich bei den SR-A- eine Notarztbegleitung von 94,5 % gegenüber 48 % bei den SR-B-Zuweisungen. Neben einem signifikant niedrigeren ISS (4,1 vs. 13,9) wiesen die SR-B-Patienten zu 29,8 % keine in der S3-Leitlinie festgelegten Schockraumkriterien auf. Bei einer Upgrade-Rate von 4,9 % konnten die SR-B-Patienten zu 98 % in sehr gutem Zustand zügig entlassen werden (GOS von 4 oder 5).

**Diskussion:**

Die vorgestellte Kategorisierung ist eine effektive und sichere Möglichkeit, die steigende Zahl der Schockraumalarmierungen ressourcenoptimiert zu bewältigen. Durch die verbesserten Alarmierungskriterien der neuen Leitlinie ist die Aufrechterhaltung dieser separaten Versorgungsstufen aber in Zukunft vermutlich nicht erforderlich.

## Abstract (en)

**Background:**

In order to continue to efficiently provide both personnel-intensive and resource-intensive care to severely injured patients, some hospitals have introduced individually differentiated systems for resuscitation room treatment. The aim of this study was to evaluate the concept of the A and B classifications in terms of practicability, indications, and potential complications at a national trauma center in Bavaria.

**Methods:**

In a retrospective study, data from resuscitation room trauma patients in the year 2020 were collected. The assignment to A and B was made by the prehospital emergency physician. Parameters such as the injury severity score (ISS), Glasgow outcome scale (GOS), upgrade rate, and the indication criteria according to the S3 guidelines were recorded. Statistical data comparisons were made using t‑tests, χ^2^-tests, or Mann-Whitney U‑tests.

**Results:**

A total of 879 resuscitation room treatments (A 473, B 406) met the inclusion criteria. It was found that 94.5% of resuscitation room A cases had physician accompaniment, compared to 48% in resuscitation room B assignments. In addition to significantly lower ISS scores (4.1 vs. 13.9), 29.8% of B patients did not meet the treatment criteria defined in the S3 guidelines. With a low upgrade rate of 4.9%, 98% of B patients had a GOS score of 4 or 5.

**Conclusion:**

The presented categorization is an effective and safe way to manage the increasing number of resuscitation room alerts in a resource-optimized manner.

## Einleitung und Hintergrund

Vor dem Hintergrund der damals aktuellen S3-Leitlinie Polytrauma 2016 [1] (LL-2016) wurde im Jahr 2018 die Empfehlung einer differenzierten Schockraumanmeldung durch den Rettungsdienstausschuss in Bayern herausgegeben [2]. Darin wurde eine bayernweite Differenzierung in einen Schockraum A (SR-A) für schwer verletzte Patienten und einen Schockraum B (SR-B) für potenziell schwer verletzte Patienten ausgesprochen. Die Definition „potenziell schwer verletzter Patient“ umfasste einen entsprechenden Unfallmechanismus und/oder die Einschätzung des präklinischen Teams ohne offensichtlich schwere Verletzungen bei stabilen Vitalparametern. Diese Empfehlung ergab sich u. a. aus der Forderung des Rettungsdienstes, potenziell schwer verletzte Patienten, äquivalent zum SR‑A, strukturiert und ohne Zeitverzug in der Klinik übergeben zu können. Die korrekte Indikationsstellung für eine Schockraumanmeldung kann präklinisch u. U. schwierig sein, und die Kriterien der Leitlinie (Tab. 1) lassen sich nicht immer eindeutig anwenden [3, 4]. Für Patienten, die keine Indikation für einen Schockraum (A) hatten, sollte der SR‑B eine neue Option darstellen. Um dem personellen und ökonomischen Druck bei gleichzeitig konstantem medizinischen Versorgungsstandard entgegenzuwirken, wird das Thema der unterschiedlichen Alarmierungsstufen immer wieder aufgegriffen [5, 6]. Die Versorgungsstufen unterscheiden sich je nach Klinik durch den Umfang des primär informierten und anwesenden Klinikpersonals. Das Ziel ist es, den Übergang zwischen Präklinik und Klinik effizient zu gestalten.

**Table 1:** 

Kriterien der SR-Alarmierung S3-LL 2016	Empfehlungsgrad	Kriterien der SR-Alarmierung S3-LL 2022	Empfehlungsgrad
*A/B – Problem* – Atemstörungen/Intubationspflicht nach Trauma *C – Problem* – Systolischer Blutdruck unter 90 mm Hg (altersadaptiert bei Kindern) nach Trauma *D – Problem* – GCS unter 9 nach Trauma	*A*	*A/B – Problem* – Atemstörungen (S_p_O_2_ < 90 %)/erforderliche Atemwegssicherung – AF < 10 oder > 29 *C – Problem* – Systolischer Blutdruck < 90 mm Hg – Herzfrequenz > 120/min – Schockindex > 0,9 – Positiver eFAST *D – Problem* – GCS ≤ 12 *E – Problem* – Hypothermie < 35,0 °C	*A*
*Verletzungen* – Instabiler Thorax – Beckenfrakturen – Vorliegen von penetrierenden Verletzungen der Rumpf-Hals-Region – Vorliegen von Schussverletzungen der Rumpf-Hals-Region – Amputationsverletzung proximal der Hände/Füße – Querschnittsverletzung – Frakturen von mehr als 2 proximalen Knochen – Offene Schädelverletzungen – Verbrennungen > 20 % und Grad ≥ 2b	*A*	*Verletzungen* – Instabiler Thorax – Mechanisch instabile Beckenverletzung – Vorliegen von penetrierenden Verletzungen der Rumpf-Hals-Region – Amputationsverletzung proximal der Hände/Füße – Sensomotorisches Defizit nach Wirbelsäulenverletzung – Prähospitale Intervention (erforderliche Atemwegssicherung, Thoraxentlastung, Katecholamingabe, Perikardiozentese, Anlage eines Tourniquets)	*A*
– Sturz aus über 3 m Höhe – Verkehrsunfall mit a) Frontalaufprall mit Intrusion von mehr als 50–75 cm, b) einer Geschwindigkeitsveränderung von Δ 30 km/h, c) Fußgänger‑/Zweiradkollision, d) Tod eines Insassen, e) Ejektion eines Insassen	*B*	*Verletzungen* – Frakturen von 2 oder mehr proximalen großen Röhrenknochen – Verbrennungen > 20 % und Grad ≥ 2b	*B*
		*Unfallmechanismus* – (Ab)Sturz aus über 3 m Höhe – Verkehrsunfall mit Ejektion aus dem Fahrzeug oder Fraktur langer Röhrenknochen	*B*
		*Geriatrische Patienten nach relevantem Trauma* – RR_sys_ < 100 mm Hg – Bekanntes oder vermutetes Schädel-Hirn-Trauma und GCS ≤ 14 – 2 oder mehr verletzte Körperregionen – Fraktur eines oder mehrerer langer Röhrenknochen nach Verkehrsunfall	*B*

*AF* Atemfrequenz, *eFAST* Extended Focused Assessment with Sonography in Trauma, *GCS* Glagow Coma Score, *S3-LL 2016* S3-Leitlinie Polytrauma/Schwerverletzten-Behandlung 2016, *S3-LL 2022* S3-Leitlinie Polytrauma/Schwerverletzten-Behandlung 2022

Insbesondere an größeren Krankenhäusern (> 500 Betten) mit einer Zertifizierung als überregionales Traumazentrum (ÜTZ) [7] und entsprechend hohen Fallzahlen kann ein abgestuftes Schockraumalarmierungs- und Behandlungsprozedere sinnvoll sein, da die Versorgung schwer verletzter Patienten sowohl personal- als auch ressourcenintensiv ist und erhebliche Vorhaltekosten verursacht [8–10]. Dass, entgegen der seit Jahren relativ konstanten Anzahl schwer verletzter Patienten, die absolute Zahl der Schockraumalarmierungen steigt, verdeutlicht die Problematik der Ressourcenbindung in allen Bereichen der Rettungskette [9, 11–13]. An der Klinik der Autoren wurde das Konzept einer abgestuften Schockraumalarmierung mit SR‑A und -B im Jahr 2019 eingeführt. Nach unserem Kenntnisstand gibt es bisher keine Analyse dieser präklinischen Differenzierung. Vor diesem Hintergrund werten wir monozentrisch und retrospektiv eine differenzierte Schockraumanmeldung hinsichtlich der Verletzungsbilder, der Verläufe und des Auftretens möglicher Komplikationen aus.

## Material und Methoden

### Schockraumkategorisierung

**Figure 1:**
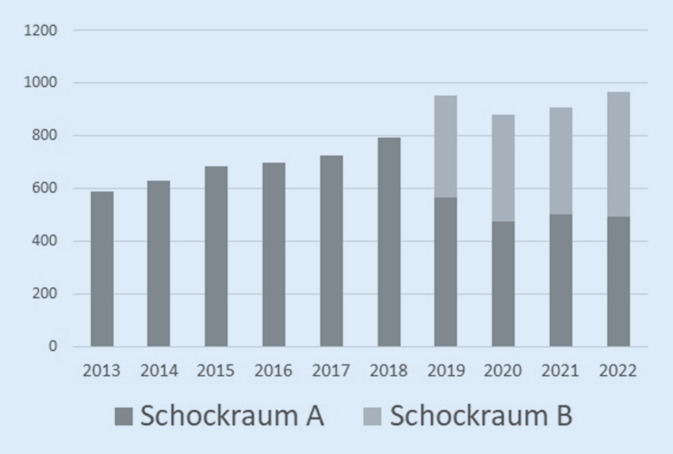

Die Zuteilung zu unterschiedlichen Alarmierungsstufen erfolgte in der hier vorgestellten Studie wie folgt: Nach Meldung des präklinischen Teams vor Ort an die integrierte Leitstelle wird die entsprechende Anmeldung eines SR‑A an das Notfalltelefon auf der anästhesiologischen Intensivstation durchgeführt, welche die weitere Alarmierung des gesamten Schockraumteams über eine Rufkette sicherstellt. Ein SR‑B wird telefonisch und elektronisch als Funkmeldung direkt an das Team der Notaufnahme gemeldet. In diesem Fall übernimmt das Team der Notaufnahme die Alarmierung des reduzierten Behandlungsteams *(SR-B-Team)*. Die kreislaufstabilen Patienten ohne offensichtliche Verletzungen werden dabei in einem separaten Schockraum – angrenzend zum eigentlichen Schockraum – durch das *SR-B-Team* in Empfang genommen. Dieses besteht aus einem Unfallchirurgen, 2 Pflegekräften und einem Radiologen. Nach Übergabe folgen die umgehende Untersuchung gemäß ATLS-Standard und primäre Bildgebung mit der Möglichkeit, das SR-A-Team jederzeit zu alarmieren (Upgrade). Die Umorganisation in SR‑A und -B erfolgte 2019 und führte bereits im Jahr der Einführung zu einer deutlichen Umverteilung (Abb. 1 und 2).

**Figure 2:**
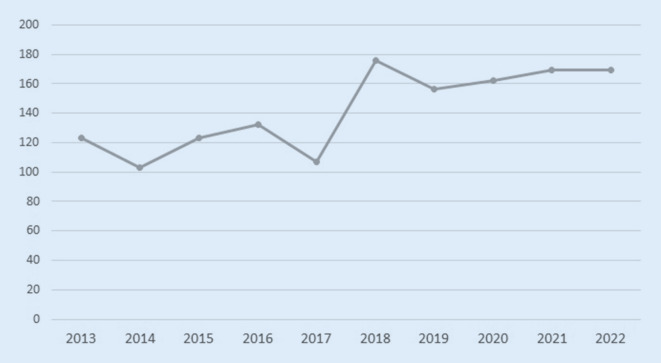

### Methodik und Studiendesign

An einem universitären ÜTZ wurde im Rahmen einer retrospektiven Studie das Kollektiv von Schockraumpatienten des Jahres 2020 evaluiert. Eine Genehmigung durch die Ethikkommission zur Auswertung der Daten liegt vor (AZ 20210205-02). Die Daten dieser Patientengruppe wurden aus dem lokalen Klinikinformationssystem (KIS i.s.h.med., Medico Fa. Cerner, DE) ermittelt und vollständig pseudonymisiert in eine nur dem Studienteam zugängige Datenbank (Excel, Fa. Microsoft Corporation, Redmond, WA, USA) eingetragen. Hierbei wurden folgende Parameter erhoben: *Alter, Geschlecht, Verletzungsmechanismus, Transportart, Injury Severity Score (ISS), Zutreffen der Schockraumkriterien nach S3-Leitlinie (LL-2016), notwendige chirurgische Intervention, Outcome (modifizierte Glasgow Outcome Scale (GOS) gemäß TraumaRegister DGU), sowie ein evtl. nötiges Upgrade zur nächsthöheren Alarmierungsstufe. *Die eingeführten unterschiedlichen Alarmierungsstufen SR‑A und -B orientierten sich zum Zeitpunkt der Analyse an der damals geltenden S3-Leitlinie Polytrauma 2016 [1]. Dementsprechend wurden potenziel schwerverletzte Patienten ohne eindeutiche Schockraumkriterien (GoR A) als Schockraum B kateogrisiert. Die Zuteilung zur jeweiligen Alarmierungsstufe erfolgt im Rahmen der Anmeldung durch den behandelnden Notarzt. Eine nachträgliche Höherstufung wurde in der Analyse nur registriert, sofern sie nach Eintreffen des Patienten erfolgte. Elementares Einschlusskriterium war die Schockraumalarmierung aufgrund eines Unfalls. Sekundärverlegungen ohne Unfall im Sinne einer weiteren chirurgischen Versorgung (z. B. Verlegung auf Intensiv, Blutung nach abdominellen Eingriffen etc.) wurden als Ausschlusskriterium gewertet. Die statistische Analyse wurde mithilfe der Statistiksoftware SPSS (IBM SPSS Statistics, Version 28.0., Fa. IBM Corp., Armonk, NY, USA) durchgeführt. Zur Analyse wurde der *t*-Test für unabhängige Stichproben oder der Mann-Whitney-U-Test verwendet. Um kategoriale Variablen zu untersuchen, wurde der χ^2^-Test benutzt. Die statistische Signifikanz wurde mit einem *p* < 0,05 festgelegt.

## Ergebnisse

Im analysierten Jahr 2020 wurden 473 Einsätze des SR‑A und 406 Einsätze im SR‑B registriert. Bei den SR-A-Fällen handelte es sich zu 72,6 % (*n* = 344) um männliche Patienten mit einer Altersverteilung von 0 bis 105 (Md: 54) Jahren. Bei den SR-B-Anmeldungen waren 266 (65,5 %) männlichen Geschlechts mit einer Altersspanne von 1 bis 109 Jahren (Md: 40).

Den Großteil der Zuweisungen als SR‑A, analysiert nach Unfallmechanismus, waren Stürze (40,8 %) und Verkehrsunfälle (38,9 %). Die übrigen Zuweisungen verteilten sich auf Suizidversuche (4,2 %) und sonstige Verletzungsarten (16,1 %). Auch im Rahmen der SR-B-Aktivierungen waren Verkehrsunfälle (56,6 %) und Stürze (30,8 %) führend, wenn auch mit größerem Unterschied.

Im Hinblick auf die Transportart (luftgebunden mit Notarzt (NA), bodengebunden ohne/mit Notarzt) lässt sich erkennen, dass signifikant häufiger eine Notarztbegleitung bei den SR-A-Zuweisungen erfolgte als bei der niedrigeren Alarmierungsstufe (*p* < 0,001).

Das Vorliegen der jeweiligen Schockraumkriterien nach der LL-2016 konnte bei den SR-A-Zuweisungen zu 85,2 % (*n* = 403) bestätigt werden, wohingegen lediglich bei 70,2 % (*n* = 208) der SR-B-Anmeldungen entsprechende Kriterien vorlagen. Demnach erfüllte ein Drittel der SR-B Patienten keinerlei Alarmierungskriterien (Tab. 1). Eine andere Möglichkeit, um diese Übertriage zu monitoren, stellen nach dem „Committee on Trauma“ des „American College of Surgeons“ (ACSCOT) [14] folgende retrospektive Kriterien dar: ein Versterben des Patienten, eine stationäre Aufnahme > 48 h, ein Aufenthalt auf einer Intensivstation oder eine notwendige Operation. Bei Anwendung dieser Kriterien zeichnet sich ein noch deutlicheres Bild ab: bei den SR-A Patienten erfüllten 93,9 % (*n* = 444) mindestens ein Kriterium und bei bei den SR‑B Patienten waren es lediglich lediglich bei 51,2 % (*n* = 208) (Tab. 2).

**Table 2:** 

–	**Alarmierungskriterien S3-Leitlinie (2016)**	
	*Trifft zu (%)*	*Trifft nicht zu (%)*
SR‑A	403 (85,2)	70 (14,8)
SR‑B	285 (70,2)	121 (29,8)
–	**Kriterien des American College of Surgeons CT**	
SR‑A	444 (93,9)	29 (6,1)
SR‑B	208 (51,2)	198 (48,8)

*SR-A* Schockraum A, *SR-B* Schockraum B, *CT* Committee on Trauma

Zudem zeigte sich, dass 82,9 % (*n* = 58) der SR-A-Patienten, die keine Anforderungskriterien der LL-2016 aufwiesen, einen signifikant niedrigen ISS unter 16 Punkten besaßen (*p* < 0,001). Unter den SR-B-Zuweisungen, die keine S3-Kriterien erfüllten, waren es sogar 99,2 % (*n* = 206), die einen ISS unter 16 Punkten hatten, allerdings ohne statistische Signifikanz (*p* = 0,09). Zur Beurteilung der Verletzungsschwere wurde der jeweilige individuelle ISS bestimmt. Die Daten beweisen eine statistisch signifikant geringere Verletzungsschwere (ISS) der Zuweisungen über den SR‑B (MW 4,1 ± 4 vs. MW 13,9 ± 14, *p* < 0,001).

Insgesamt wurden 11 Patienten (2,7 %) nach Klinikeintreffen in die höhere Alarmierungsstufe gruppiert (Tab. 3). Fünf der hochgestuften Patienten (45,5 %) wiesen einen ISS von mehr als 16 Punkten auf. Insgesamt zeigte sich ein signifikanter Unterschied hinsichtlich der Verletzungsschwere (ISS) zwischen den hochgestuften und verbliebenen SR-B-Patienten (Md. 13 vs. Md. 2, *p* < 0,001). Die unterschiedlichen Ursachen für eine Höherstufung sind Hypotension, Vigilanzminderung, Desaturation und interventionsbedürftige Diagnose nach Primärdiagnostik. Außerdem zeigte sich, dass die hochgestuften Patienten signifikant älter waren (Md 61 vs. 39, *p* < 0,001). Trotz des Zeitverzugs durch eine Höherstufung erst nach Klinikeintreffen, lag der durchschnittliche Wert der GOS bei 4 Punkten. Die zwei registrierten Todesfälle nach initialer Zuweisung als SR‑B ereigneten sich im Rahmen der operativen Intervention bzw. des Intensivaufenthalts im weiteren Verlauf und ergaben keinen Hinweis auf einen direkten Zusammenhang zur Alarmierungsstufe.

**Table 3:** 

Sex	Alter	Unfallmechanismus	Transport	Upgrade-Ursache	Verletzte Region	ISS	Outcome (mod. GOS)
♂	88	Sturz	RTW + NA	Vigilanz ↓	SHT (Mono)	9	3
♂	95	Sturz	RTW + NA	Vigilanz ↓	SHT (Mono)	16	1
♀	55	VU, Fahrrad	RTW + NA	Vigilanz ↓	SHT (Mono)	3	5
♂	61	Sonstiges	RTH	Hypotension	Untere Extremität (Mono)	9	5
♀	82	Suizid	RTW + A	Hypotension	Wirbelsäule (Mehrfach)	21	1
♂	39	Sturz	RTW + NA	Hypotension	Thorax (Mono)	25	5
♂	61	Sonstiges	RTW	Hypotension	Obere Extremität (Mono)	0	5
♂	50	VU, Motorrad	RTW + NA	Sauerstoffsättigung ↓	Thorax (Mehrfach)	13	5
♂	56	VU, Pkw	RTH	Sauerstoffsättigung ↓	Thorax (Mehrfach)	8	5
♂	80	VU, Pkw	RTW + NA	V. a. freie abdominelle Flüssigkeit	Thorax/Abdomen	18	5
♂	69	Sturz	RTH	V. a. freie abdominelle Flüssigkeit	Thorax/Wirbelsäule	22	4

*VU* Verkehrsunfall, *RTW* Rettungswagen, *NA* Notarzt, *V.a.* Verdacht auf, *GOS* Glagow Outcome Score, *ISS* Injury Severity Score

Eine unmittelbare chirurgische Intervention, sei es direkt im Schockraum im Sinne einer Thoraxdrainage oder eines Major-Eingriffs im Zentral-OP innerhalb der ersten 6 h nach Eintreffen, musste signifikant häufiger bei den SR-A-Zuweisungen durchgeführt werden (*p* < 0,001). Hier waren es 39,5 % (*n* = 187) gegenüber 18,5 % (*n* = 77).

Das ermittelte Outcome der SR-A-Patienten lag zu 76,4 % auf den beiden höchsten Stufen 5 („gut erholt“) und 4 *(„mäßig behindert“)*. Bei 58 Patienten (12,2 %) trat entweder im Schockraum oder während der nachfolgenden Intensivbetreuung der Tod ein. Im Gegensatz dazu erreichten 97,8 % der als SR‑B eingestuften Patienten die beiden höchsten Stufen nach GOS, wobei 6 Patienten (1,5 %) ihren Verletzungen erlagen (Tab. 4).

**Table 4:** 

	Schockraum A (%)	Schockraum B (%)	*p*-Wert
*Einsätze*	473	406	–
*Geschlecht*			< 0,001
Männlich	344 (72,6)	266 (65,5)	
Weiblich	129 (27,4)	140 (34,5)	
*Alter*	54 (31–70) Median (IQR)	40 (22–59) Median (IQR)	< 0,001
*Unfallmechanismus*			< 0,001
Verkehrsunfall	184 (38,9)	230 (56,6)	
Sturzereignis	193 (40,8)	125 (30,8)	
Suizid	20 (4,2)	7 (1,7)	
Sonstiges	76 (16,1)	44 (10,9)	
*Transportart*			< 0,001
Unbekannt	18 (3,8)	120 (29,6)	
RTW	8 (1,7)	90 (22,2)	
RTW + Notarzt	183 (38,7)	113 (27,8)	
RTH	264 (55,8)	83 (20,4)	
*Injury Severity Score*			
≥ 16	169 (35,7)	12 (2,9)	< 0,001
Median ISS	9 (4–18) Median (IQR)	2 (1–5) Median (IQR)	< 0,001
*Chirurgische Intervention*	187 (39,5)	77 (18,9)	< 0,001
*Outcome (mod. GOS)*			< 0,001
5	207 (43,9)	332 (81,8)	
4	155 (32,8)	65 (16,0)	
3	42 (8,7)	3 (0,7)	
2	12 (2,5)	–	
1	57 (12,1)	6 (1,5)	

*RTW* Rettungswagen, *RTH* Rettungshubschrauber

## Diskussion

Effizient strukturierte Arbeitsabläufe sind elementarer Bestandteil der Sicherstellung einer funktionsfähigen Notfallversorgung. Die hier vorgestellte Kategorisierung ist eine Möglichkeit, den Patientenzustrom weiter zu differenzieren und vorhandene Ressourcen effizient zu nutzen. Die ähnlich hohen Fallzahlen im SR‑A (*n* = 473) und SR‑B (*n* = 406) zeigen den Effekt dieser in Bayern regional unterschiedlich etablierten Unterteilung.

Die 2011 veröffentlichte und 2016 überarbeitete S3-Leitlinie zur Versorgung schwer verletzter Patienten [1] legitimierte die Schockraumalarmierung anhand des Unfallmechanismus. In den vorgestellten Daten lag ein Verkehrsunfall in mehr als der Hälfte der SR-B-Zuweisungen (56,6 %) vor. Bereits in der damaligen Langfassung der Leitlinie werden Studien zitiert, die über Raten der Fehleinschätzung auf Basis des Unfallmechanismus bis zu 92 % berichten [15]. Dass der Verletzungsmechanismus per se ein Verdachtsmoment rechtfertigt, ist selbstverständlich [16]. Allerdings sind Faktoren wie die Geschwindigkeitsdifferenz unzuverlässig quantifizierbar und mittlerweile obsolet [3]. Die überarbeite Leitlinie 2022 (LL-2022) berücksichtigt diesen Sachverhalt und führt nur noch „Sturz aus über 3 m Höhe“ sowie „Verkehrsunfall mit Ejektion aus dem Fahrzeug oder Fraktur langer Röhrenknochen“ als Schockraumalarmierungskriterium auf [17]. Zur Begründung wird mit einer Konsensstärke von 100 % eine Studie von Dehli et al. mit Daten aus den Jahren 2013 und 2014 herangezogen. Trotz der geringen Fallzahlen konnten die Kollegen zeigen, dass bei 66 % der aus einem Fahrzeug geschleuderten Patienten (*n* = 4) und bei 50 % der aus über 5 m Höhe gestürzten (*n* = 10) ein ISS > 15 Punkten vorlag [18]. Allerdings beschreiben sie ebenfalls, dass bei diesen Patienten noch zusätzliche Kriterien vorlagen, die eine Schockraumaktivierung neben dem Unfallmechanismus indizierten. Deshalb ist eine alleinige Verwendung dieser beiden Unfallmechanismen als Schockraumindikation sicherlich selten. Auch international hat der alleinige Unfallmechanismus eine untergeordnete Rolle. In den „*Guidelines for Field Triage der ACSCOT*“ (2011), die letztendlich auch Einfluss auf die S3-Leitlinie nehmen, werden die unfallbezogenen Mechanismen als Step-3-Kriterien bezeichnet und rechtfertigen bei alleinigem Vorliegen keinen Transport in das Traumzentrum der höchsten Stufe [22]. Auch in unseren Daten spiegelt sich mit einem durchschnittlichen ISS von 4 Punkten (SR-B) die geringe Verletzungsschwere der nach Unfallhergang eingelieferten Patienten wider.

Nachdem die Daten der veralteten LL-2016 zum Thema Schockraumalarmierung teilweise bis in die 1970er-Jahre zurückreichen, wurde dieser Teil in der am 31.12.2022 neu erschienenen S3-Leitlinie zur Polytrauma/Schwerverletzten-Behandlung komplett überarbeitet [17]. Als Kriterien zur Schockraumaktivierung werden nun entweder klinische Parameter oder bestimmte Verletzungsmuster sowie prähospitale Interventionen aufgeführt (Tab. 1). Während vormals verstärkt Verletzungsmuster gewertet wurden, gibt die neue Leitlinie nun auch eine große Anzahl klinischer Parameter nach Trauma vor, deren Auftreten mit einer erhöhten Mortalität verknüpft ist [19, 20]. Die Durchsetzung in der Präklinik und ein daraus resultierender Effekt müssen abgewartet werden. Eine gewisse Dynamik ist zu erwarten, da zukünftig die Indikationsstellung für geriatrische Patienten zur Schockraumanmeldung deutlich ausgeweitet wurde [17]. Auch in unseren Daten spiegelt sich im höheren Durchschnittsalter bei den via SR‑A zugewiesenen Patienten die steigende Bedeutung des Patientenalters wider.

Das Konzept eines reduzierten Einsatzteams (Schockraum B, Schockraum light o. Ä.) steht auch in der Kritik einer möglichen Unterschätzung, was für alle Beteiligten schwerwiegende Konsequenzen bedeuten kann. Nach ACSCOT definiert eine solche Fehleinschätzung formal den Transport eines Schwerverletzten in ein nicht dafür ausgestattetes Traumazentrum. Diese Rate sollte nicht mehr als 5 % betragen [14]. In der hier vorgestellten Studie wurden SR-B Patienten nach Upgrade auf Schockraum A als fehlplatziert definitiert. Da die umfangreiche Diagnostik und Versorgung jederzeit gewährleistet sind, jedoch im Falle eines Upgrades mit wenigen Minuten Verzögerung, gibt es hierzu in der Literatur keine vergleichbaren Empfehlungen. Die Upgrade-Rate von 2,7 % weist jedoch auf eine durchaus gute präklinische Einschätzung hin.

Neben der Unterschätzung kann eine erhöhte Überschätzung (> 25–35 %) ebenfalls negative Auswirkungen haben, auch wenn die Konsequenzen anders als bei einer fallbasierten Untertriage schwerer messbar sind und zeitverzögert auftreten. Bei einer Schockraumalarmierung ist die Ressourcenbindung erheblich, und insbesondere außerhalb der Kernarbeitszeit kommen andere Arbeitsprozesse teilweise vollständig zum Stillstand. In diesem Sinne kann die Einführung unterschiedlicher Alarmierungsstufen anhand der damals geltenden Kriterien rückwirkend als durchaus sinnvoll erachtet werden, um eine systemlähmende Übertriage mit inadäquaten Schockraumzuweisungen zu verhindern. *Auch im Hinblick darauf, dass bei fast 30* *% aller SR-B-Einlieferungen keines der ehemals in der LL-2016 beschriebenen Kriterien vorlag, die eine Schockraumindikation rechtfertigen.*

## Limitationen

Limitationen sind das retrospektive Studiendesign, ein begrenzter Beobachtungszeitraum teilweise noch unter Pandemiebedingung, die isolierte Betrachtung eines Versorgers und die kleine Kohorte. Für die zukünftige Entwicklung ist festzuhalten, dass bei konsequenter Anwendung der weit gefassten Alarmierungskriterien der neuen Leitlinie (LL2022) sich nur wenige Indikationen für einen Schockraum B ergeben, was die Beibehaltung dieses Konzepts langfristig infrage stellt [21]. Der Begriff Schockraum sollte dann entsprechenden Fällen vorbehalten sein.

## Fazit für die Praxis

- Durch differenzierte Schockraumalarmierungskriterien war ein effizienter Einsatz des Behandlungsteams möglich.
- Abgestufte Einsatzkapazitäten könnten ein hilfreiches Mittel für eine Ressourcenschonung ohne Patientengefährdung sein.
- Bei konsequenter Anwendung der neuen Leitlinie (LL2022) werden sich in Zukunft wenige Indikationen für ein abgestuftes Versorgungskonzept ergeben.
- Insofern sollte aus unserer Sicht die Kategorie Schockraum entsprechend der LL2022 gestärkt werden. Die Aufrechterhaltung einer abgestuften Schockraumvariante ist allerdings zu diskutieren.
